# A novel antibody combination to identify KIR2DS2^high^ natural killer cells in KIR2DL3/L2/S2 heterozygous donors

**DOI:** 10.1111/tan.13413

**Published:** 2019-01-15

**Authors:** Matthew D. Blunt, Pauline Rettman, Leidy Y. Bastidas‐Legarda, Rebecca Fulton, Valentina Capizzuto, Mohammed M. Naiyer, James A. Traherne, Salim I. Khakoo

**Affiliations:** ^1^ Clinical and Experimental Sciences Faculty of Medicine, University of Southampton, Southampton General Hospital Southampton UK; ^2^ Division of Immunology, Department of Pathology University of Cambridge Cambridge UK

**Keywords:** antibody, KIR, KIR2DS2, NK cells

## Abstract

The killer cell immunoglobulin‐like receptor (KIR) KIR2DS2 induces natural killer (NK) cell activation upon ligation and in genetic studies is associated with protection against certain cancers and viral infections. One of the difficulties in understanding KIR2DS2 has been that ligands have been hard to define. In part, this is because the high sequence homology between KIR2DS2 and KIR2DL3/KIR2DL2 has made it difficult to make antibodies that specifically detect NK cells expressing KIR2DS2. Using transfected NK cell line (NKL) cells and primary human samples, we report the identification of a novel antibody combination which allows identification of NK cells with relatively high expression of KIR2DS2. This separation is sufficient to examine primary human NK cell activation in response to KIR2DS2 specific ligands.

Natural killer (NK) cells are controlled by an array of activating and inhibitory receptors and are important for clearing cancer and virus‐infected cells.^1^, ^2^ The killer cell immunoglobulin‐like receptors (KIRs) are expressed by NK cells as well as subsets of T cells and recognise the combination of HLA‐C with bound peptide. The specificity of inhibitory KIRs have been well documented, as they detect HLA‐C downregulation and are also modulated by changes in the peptide content of HLA‐C.^3^, ^4^, ^5^ In contrast, much less is known about the activating KIR, although they have been shown to play a significant role in viral infections and cancer.^6^, ^7^, ^8^, ^9^ The activating KIR, KIR2DS2, is of particular interest given its protective role in bone marrow and cord blood transplantation for various hematological malignancies^10^, ^11^ and in glioblastoma models in vivo.^12^ In addition, KIR2DS2 has recently been shown to directly recognise viral helicase peptides in the context of HLA‐C and to be protective against chronic hepatitis C virus infection.^9^ One of the barriers to understanding KIR2DS2 has been that, the high sequence homology between KIR2DS2 and the inhibitory KIR, KIR2DL2 and KIR2DL3, makes it difficult to develop antibodies that specifically detect KIR2DS2. In this study, we sought to identify an antibody combination, which could discriminate KIR2DS2 from both KIR2DL2 and KIR2DL3.

Antibodies to detect KIR2DS2 positive NK cells in primary human samples have previously been described. However, these are only able to detect KIR2DS2 expression in donors with particular KIR genotypes, or have been unable to discriminate between KIR2DS2 and the inhibitory KIRs KIR2DL2 and KIR2DL3. For example, the antibodies CH‐L, DX27 and GL‐183 detect KIR2DS2, but also detect KIR2DL2 and KIR2DL3.^13^, ^14^, ^15^, ^16^ The antibody clone 1F12 is able to distinguish KIR2DS2 from KIR2DL2, but also binds KIR2DL3 and can therefore only be used in donors with a KIR2DL2/KIR2DS2 homozygous genotype, who lack KIR2DL3.^17^

We utilised NKL cell lines transfected with KIR2DL2, KIR2DL3 or KIR2DS2 to probe the specificity of the antibody clones CH‐L (BD Biosciences, Wokingham, UK) and REA147 (Miltenyi Biotech, Woking, UK) for each KIR of interest. Antibody clone CH‐L was found to bind KIR2DL2, KIR2DL3 and KIR2DS2 (Figure 1A), as previously reported. In contrast, we found binding of antibody clone REA147 to KIR2DL2 and KIR2DL3, but no significant binding to KIR2DS2 (Figure 1A). We therefore hypothesised that the combination of antibody clones CH‐L and REA147 could be used to discriminate primary human NK cells which express high levels of KIR2DL3/KIR2DL2 from those which express predominantly higher levels of KIR2DS2 (CH‐L positive and REA147 negative). We tested 21 donors of various KIR2DL3/2DL2/2DS2 genotypes (assessed by PCR using sequence‐specific primers^18^). Overall these donors gave three distinct staining patterns that distinguished the different KIR genotypes; KIR2DL3 homozygous, KIR2DL2/S2 homozygous and KIR2DL3/L2/S2 heterozygous (Figure 1B). NK cells from KIR2DL3 homozygous donors showed a KIR2DL3 positive population, identified as double positive for CH‐L and REA147. NK cells from KIR2DL2/KIR2DS2 homozygous donors had a population positive for CH‐L which could be divided based on positive or negative expression of REA147 (Figure 1B). This is indicative of relatively higher KIR2DL2 expression (KIR2DL2^high^) or relatively higher expression of KIR2DS2 (KIR2DS2^high^) in these populations. Finally, KIR2DL3/KIR2DL2/KIR2DS2 heterozygous donors showed a combination of the two previously described phenotypes. There were distinct double CH‐L and REA147 positive populations similar to that seen in KIR2DL3 homozygous donors, and also a population positive for CH‐L, which could be divided based on REA147 positivity into KIR2DL3/L2^high^ or KIR2DS2^high^ NK cells (Figure 1B). No differences in staining pattern were evident for the allelic variants present in this cohort for *KIR2DL3* (*001,*002), *KIR2DL2* (*001,*003) or *KIR2DS2* (*001,*006). A full list of the allelic typing data is given in Table S1, Supporting Information.

**Figure 1:**
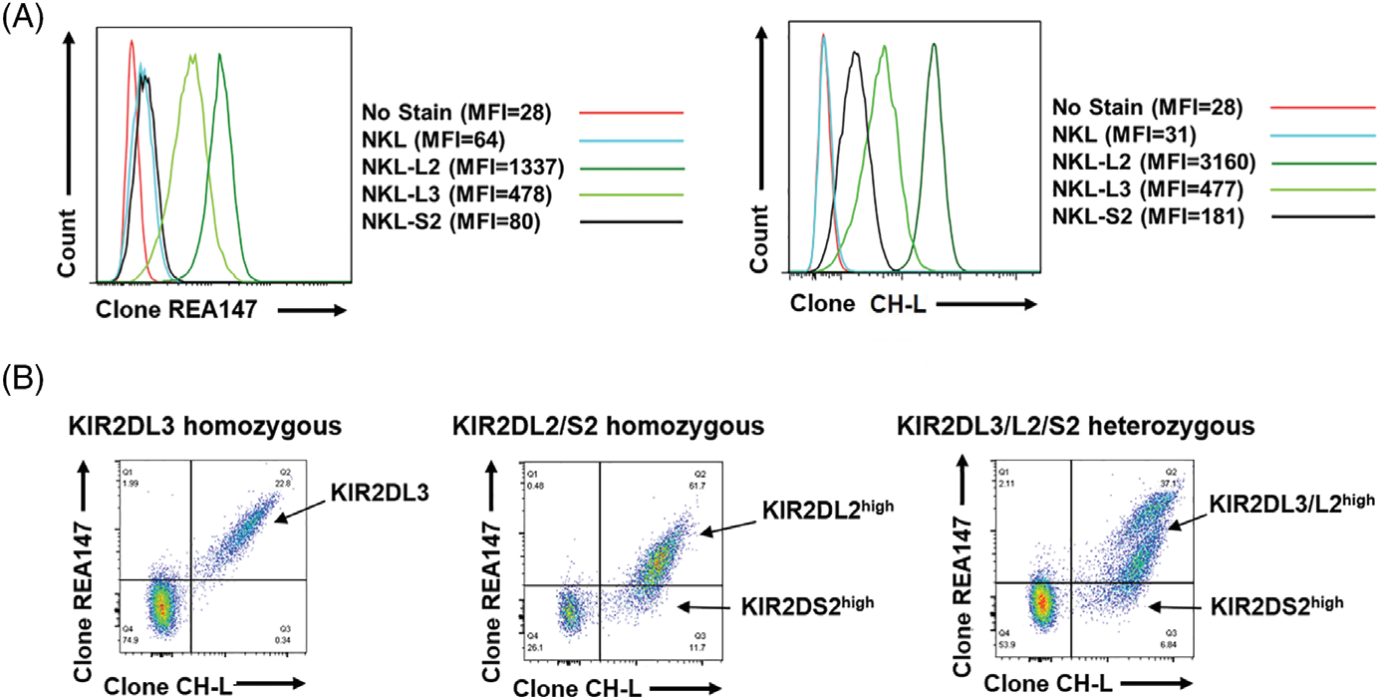
Discrimination of KIR2DS2 from KIR2DL2 and KIR2DL3. A, NKL cell lines either untransfected or transfected with KIR2DL2, KIR2DL3 or KIR2DS2 were stained with antibody clone REA147‐FITC at 1:10 dilution (Miltenyi Biotech) or antibody clone CH‐L‐PE at 4:100 dilution (BD Biosciences) and analysed by flow cytometry. B, Representative flow cytometry plots of primary human CD3‐CD56+ natural killer (NK) cells stained with REA147 and CH‐L are shown from KIR2DL3 homozygous (of seven donors), KIR2DL2/KIR2DS2 homozygous (of four donors) and KIR2DL3/KIR2DL2/KIR2DS2 heterozygous (of 10 donors) donors (assessed by PCR using sequence specific primers^18^). The KIR2DL3/L2^high^ and KIR2DS2^high^ NK cell populations detected by flow cytometry are indicated

To test whether this antibody combination would identify primary human NK cells with a predominantly activating KIR2DS2 function, we tested viral helicase peptides which we have recently shown to specifically bind and stimulate KIR2DS2.^9^ The viral helicase peptide LNPSVAATL (LNP) derived from hepatitis C virus and the IVDLMCHATF (IVD) peptide derived from dengue virus both bind HLA‐C*0102 and induce binding of KIR2DS2 to HLA‐C*0102. Both peptides share the conserved ‘AT’ motif at the C‐terminal −1 and − 2 positions, which is crucial for KIR2DS2 recognition. We used the MHC class I‐negative 721.221 transfectant expressing HLA‐C*0102 in combination with the LNP or IVD peptides as previously described.^9^ We found that both cell lines induced primary human NK cell degranulation in the putative KIR2DS2^high^ population (Figure 2A, B). In contrast, no effect of LNP or IVD was seen on degranulation in the KIR2DL3/L2^high^ NK cell population (Figure 2A,B). Furthermore, KIR2DL3 positive NK cells from KIR2DL3 homozygous donors showed no significant change in degranulation in response to either LNP or IVD stimulation (Figure 2C,D). These results indicate that this novel antibody combination is sufficient to identify a KIR2DS2^high^ NK cell population to allow detection of NK cell activation in response to specific ligands for KIR2DS2. These data provide further support using primary human NK cells that KIR2DS2 is an antigen‐specific receptor for conserved flaviviral helicase peptides.

**Figure 2:**
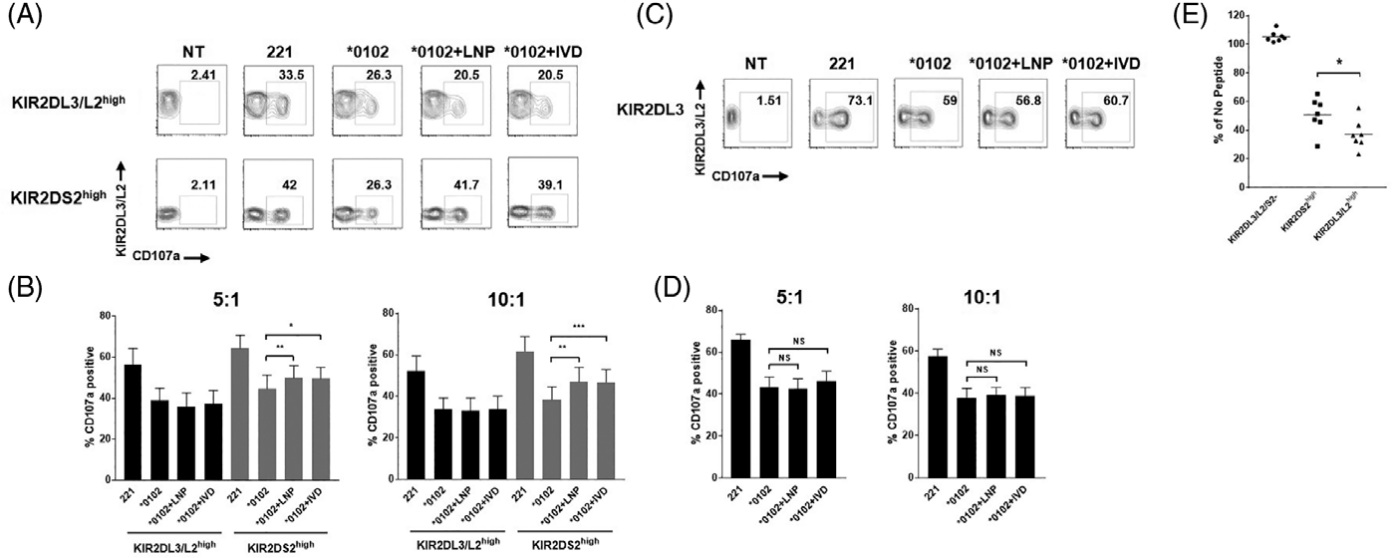
CH‐L and REA147 identify KIR2DS2^high^ natural killer (NK) cells that are activated by LNP and IVD. A, Peripheral blood mononuclear cells (PBMCs) were stimulated overnight with IL‐15 (1 ng/mL) and incubated for 4 hours with no target (NT) or 721.221 target cells either untransfected or transfected with HLA‐C*0102, HLA‐C*0102 + LNP or HLA‐C*0102 + IVD as described by Naiyer et al.^9^ CD3− CD56+ KIR2DL3/L2^high^ or KIR2DS2^high^ cells were assessed for degranulation using CD107a expression by flow cytometry. A representative flow cytometry plot from a KIR2DL3/KIR2DL2/KIR2DS2 heterozygous donor is shown in (A), at a 10:1 effector: Target ratio. Data from seven donors is shown in (B) at effector: Target ratios of 10:1 and 5:1. Mean CD107a expression ± SEM is shown. C, Degranulation of CD3− CD56+ KIR2DL3 positive cells from a representative KIR2DL3 homozygous donor as identified in Figure 1B. Summarised data for seven donors is shown in (D) at 10:1 and 5:1 effector: Target ratios. Mean CD107a expression ± SEM is shown. E, PBMCs were stimulated overnight with IL‐15 (1 ng/mL) and incubated for 4 hours with 721.174 target cells loaded with either VAPWNSFAL peptide (5 μM) or no peptide at a 5:1 effector: Target ratio. KIR2DL3/L2/S2− (negative), KIR2DL3/L2^high^ or KIR2DS2^high^, CD3− CD56+ NK cells were assessed for CD107a expression by flow cytometry. The % CD107a expression compared with “no peptide” control for seven independent experiments are shown. *P* values were determined by paired two‐tailed *t* tests (*P < 0.05, **P < 0.01, ***P < 0.001)

To test the inhibitory capacity of the identified KIR2DL3/L2^high^ and KIR2DS2^high^ populations further, we tested activation of NK cells against TAP‐deficient 721.174 target cells loaded with an inhibitory peptide for KIR2DL2 and KIR2DL3, VAPWNSFAL (VAP‐FA).^3^ Figure 2E shows that VAP‐FA inhibited activation of NK cells in the KIR2DL3/L2^high^ population with greater potency than NK cells in the KIR2DS2^high^ population. No effect of VAP‐FA was seen on the KIR2DL3/L2/S2 negative population. These results indicate that the inhibitory KIRs KIR2DL2 and/or KIR2DL3 are co‐expressed in the KIR2DS2^high^ population. However, this expression is at a lower level compared with the KIR2DL3/L2^high^ population, such that VAP‐FA has a weaker inhibitory effect. KIR2DS2+/KIR2DL2‐ NK cell clones have previously been identified.^9^, ^19^ Therefore, the KIR2DS2^high^ population identified in this study may contain a mixed population of KIR2DS2+/KIR2DL3/2+ NK cells in addition to KIR2DS2+/KIR2DL3/L2‐ NK cells. Furthermore, 721.221 cells expressing HLA‐C*0304 have previously been shown to inhibit double positive KIR2DS2+/KIR2DL2+ NK cells.^19^ In agreement with this, HLA‐C*0102 expression in the 721.221 cell line inhibited degranulation in both the KIR2DS2^high^ and KIR2DL3/L2^high^ NK cell populations (Figure 2A,B). This provides further evidence of the dominant effect of inhibitory over activatory signalling in NK cells. However, the presence of a KIR2DS2 binding peptide in conjunction with HLA‐C*0102 activates the KIR2DS2^high^ population compared with HLA‐C*0102 alone (Figure 2A,B).

We describe a novel means with which to probe the role of KIR2DS2 in primary human samples which can be utilised in donors with the KIR2DL3/L2/S2 genotype. We also provide proof‐of‐concept data that KIR2DS2‐positive NK cells recognise peptides from viral helicases. Given the increasingly recognised role of KIR2DS2 in cancer^11^, ^12^, ^20^ and viral infections,^9^ novel methods with which to investigate KIR2DS2 function in primary human NK cells should prove useful reagents for the KIR community.

## ACKOWLEDGMENTS

This work was supported by a grant from the Medical Research Council (MR/M019829/1 to S.I.K). The project has received funding from the European Research Council (ERC) under the European Union's Horizon 2020 research and innovation programme (grant agreement No. 695551).

## CONFLICT OF INTEREST

S.I.K. and M.M.N. have applied for a patent for the use of peptides for NK cell therapy. The authors have declared no conflicting interests.

### Author Contributions

M.D.B. and S.I.K. designed the experiments, analysed the data and wrote the manuscript. M.D.B, P.R, L.Y.B., R.F., V.C, J.A.T. and M.M.N. performed the research.

## Supporting information


**Table S1** Allelic typing of donors used in this study. N/A indicates data not available.
