# Supplementary material for: A novel antibody combination to identify KIR2DS2high natural killer cells in KIR2DL3/L2/S2 heterozygous donors
Source: HLA. 2019 Jan 15;93(1):32–5. doi: 10.1111/tan.13413 (PMC6492158; doi:10.1111/tan.13413)
Supplement: Supplementary file 1 — Table S1 Allelic typing of donors used in this study. N/A indicates data not available. [file TAN-93-32-s001.pdf]

Supplementary Table 1. Allelic typing of donors used in this study. N/A indicates data not available.

**Donor**	**KIR2DL3**	**KIR2DL2**	**KIR2DS2**
1	**002*	**003*	**001*
2	**002*	**003*	**001*
3	**001*	**003*	**001*
4	Negative	**001* + **003*	**001* + **001*
5	**002*	**001*	**001*
6	**001*	**001*	**006*
7	**001*	**003*	**001*
8	**001* + **001*	Negative	Negative
9	N/A	Negative	Negative
10	**001* + **001*	Negative	Negative
11	**001* + **002*	Negative	Negative
12	N/A	Negative	Negative
13	**001* + **001*	Negative	Negative
14	**001* + **001*	Negative	Negative
15	Negative	**001* + **003*	**001* + **001*
16	Negative	**001* + **003*	**001* + **001*
17	Negative	N/A	N/A
18	**001*	**003*	**001*
19	**002*	**001*	**001*
20	**001*	**001*	**001*
21	**001*	**003*	**001*
